# Transforming aquaculture with insect-based feed: restraining factors

**DOI:** 10.1093/af/vfae011

**Published:** 2024-09-05

**Authors:** Vimal Selvaraj, Eugene Won

**Affiliations:** Department of Animal Science, College of Agriculture and Life Sciences, Cornell University, Ithaca NY 14850, USA; Department of Animal Science, College of Agriculture and Life Sciences, Cornell University, Ithaca NY 14850, USA

**Keywords:** Insect, protein, fish, feed, waste, economy, industry

**Implications**

- Insect farming presents a sustainable solution for aquaculture feed, with a potential to transform feed production in the face of global food security challenges.
- The sector faces challenges in economically scaling production, ensuring safety, and formulating regulations, pointing to the need for strategic solutions.
- The article calls for focused research, investment, and innovation in insect-based feeds to meet aquafeed demands and maximize its environmental benefits.

The first explorations into utilizing insects as a food source due to the foresight of impending food demand occurred in the 1930s, the period between the world wars (Bodenheimer, 1951). By the 1980s, more structured research underscored the tangible advantages of incorporating insect larvae into animal and aquaculture feeds (Newton et al., 1977; Bondari and Sheppard, 1981, 1987). Nonetheless, the progression of applications from these investigations was overshadowed at the time by abundant, high-quality protein from marine sources (Green, 2016). Fishmeal dominated the feed industry and became the primary protein source for various farm animal diets. In 1980, fishmeal production, totaling 5.8 million metric tons, was predominantly allocated to feed terrestrial livestock, with poultry (49.8%) and swine (36.1%) being the major consumers. As wild marine fisheries reached their limits in the following decade, however, aquaculture—along with its demand for protein feed—rapidly grew to fill ever-expanding seafood markets. Aquaculture surpassed beef production in 2012 and, in 2020, the human population consumed more farmed fish (87.3 million metric tons) compared to wild-caught fish (70 million metric tons). In this same year, the aquaculture industry consumed 85% of the approximately 16 million metric tons of fish being processed for meal. [All fishmeal data from (FAO, 2022)].

With increasing fishmeal demand, several studies have highlighted a forage fish stock crisis unfolding due to overfishing as early as in the turn of the century (Worm et al., 2006; Costello et al., 2012). Today, fishmeal production remains unpredictable due to lower sustainable yields, variable environmental and climatic conditions, increasing fuel costs per tonnage of catch, and reduced quotas. For example, fishmeal supply in 2023 was 23% less than in 2022 due to a combination of factors (IFFO, 2024). In addition to the environmental controversy around fishmeal, the unpredictable volatility and overall rise in fishmeal prices has led to increased interest in alternative ingredients; however, shortcomings in plant-based proteins (e.g., soy), especially for high-value carnivorous fish like salmon, have opened the pathway for aquafeed ingredients—or at least protein supplements—with more appropriate nutritional value, with insect meals among the most highly anticipated. Offering low land and water requirements alongside high feed-to-insect biomass conversion efficiency, insect farming has been touted as a sustainable protein source for animal feeds, namely as an alternative to conventional fishmeal and soy.

Insects are viewed favorably for their potential to reduce the environmental impact of aquaculture production by using existing organic waste streams as their main input. This aspect of insect production has been modeled as part of a circular economy for addressing food system challenges, including a lifecycle that could salvage nutrients from organic industrial byproducts, or redirect population-concentrated urban wastes towards enriching rural production (Figure 1). However, as the race for market share is on, both areas of concentration and competition are diverse with various large-scale and small-scale insect manufacturers undertaking different strategies to expand their businesses at the present time.

**Figure 1.**
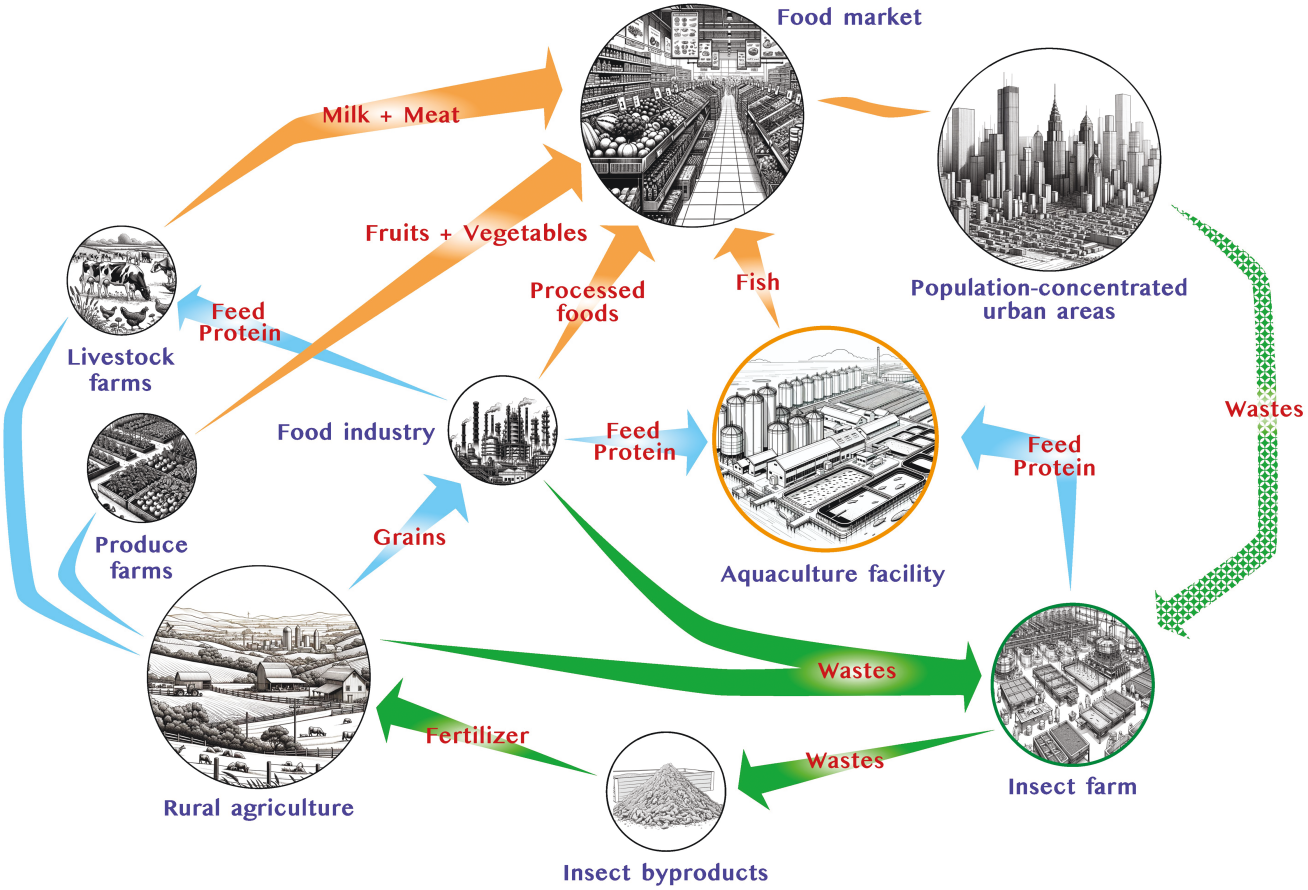
Insect-based feed industry integrated within a circular economy. This diagram illustrates an innovative insect protein production facility at the core of a circular economy model, efficiently utilizing urban and farm waste. In transforming underutilized resources into high-quality protein for aquaculture, and byproducts as fertilizer. This sustainable process can indirectly support the production of milk, meat, fruits, and vegetables, while generating additional revenue streams and mitigating waste disposal costs. This model demonstrates the feasibility and highlights the synergistic contribution to the food supply of human populations. Arrows indicate flow of agricultural/industrial products (blue); waste streams (green); consumer goods (orange).

The FAO has endorsed insect farming as meeting the global demand for sustainable and environmentally acceptable protein for animal feeds and human food (van Huis et al., 2013). Different types of insects are already competing for a share in the aquafeed market. These include black soldier flies (BSF; *Hermetia illucens*), yellow mealworms (*Tenebrio molitor*), lesser mealworms (*Alphitobius diaperinus*), silkworms (*Bombyx mori*), locusts (*Locusta migratoria* and *Schistocerca gregaria*), crickets (*Acheta domesticus*, *Gryllodes sigillatus*, and *Gryllus assimilis*), and houseflies (*Musca domestica*). Among these, large-scale manufacturing is already in the pipeline for BSF and mealworms, which are considered the most versatile and efficacious for recycling waste materials into a commodity suitable for use in aquafeeds (van Huis, 2020).

There is no question about the fit for insect-based feed in aquaculture; performance and benefits have been extensively reviewed in recent literature with a core message being that insect meal can supplant substantial rates of soy and fishmeal in aquafeeds, with results contingent on the insect meal production methods and the fish cultivar in question. The relative cost of insect meals nevertheless remains a factor yet to be determined until economies of scale can be assessed. With insect meal production just entering the industrialization stage, several issues still need to be addressed to determine if this commodity can be both economically and environmentally viable, including mass production scale, consistency of product quality, recognizing the full scope of products/byproducts and their markets, and regulatory challenges.

## Restraining factors

### Costs to scaling-up quantity

Economies of scale suggest that large facilities and automation will be needed to cost-effectively generate enough insect meal to impact the aquafeed market. Moreover, it needs to compete with the price point between soy meal ($570/metric ton; IndexMundi, end 2023), an inexpensive plant-based protein, and fishmeal ($1,870/metric ton), a high-quality animal protein; the latter being typically used at lower rates to supplement nutritional shortcomings of plant-based ingredients, especially for carnivorous fish. Currently, BSF meal is at a non-competitive price point between $2,000 and $5,000/metric ton (from industry communication).

Price notwithstanding, if the current volume of fishmeal used in aquafeed production represents a competitive market share opportunity for insect meal, then insect producers could arguably vie for about 4-5 million metric tons of product [about 85% of the 6 million metric tons of processed fishmeal produced in recent years; (IFFO, 2024)]. However, the largest operational BSF production facility claims only about 15,000 metric tons of annual BSF meal production. So, even with planned facilities projecting three to four times that volume, production levels over the next decade may still be too modest to tangibly displace fishmeal demand from aquaculture, as almost 100 insect facilities at the scale of 50,000 metric tons per year would be needed to meet market needs represented by the current volume of fishmeal. Given this metric, the scope of insect meal can be toward reducing fishmeal use by supplementation. However, even for inclusion at 10%, larva meal would require the scale up for 500,000 metric tons/year in production. Aquaculture may also need to compete with other markets open to using insect products, including other animal feed industries and pet foods, that would also need to be considered in demand projections.

Furthermore, at an estimated 5% conversion of food stock into insect meal in BSF cultivation, industries would need access to ~100 million metric tons of organic waste substrate to achieve production at levels comparable to the volume of fishmeal used for aquafeeds at the present time. To address the need for economical feedstock, the industry needs to be gravitating toward a model of planning construction of insect production facilities in proximity to massive organic waste streams, to avoid resource input bottlenecks while decreasing transportation costs and carbon footprint.

To effectively scale up insect production, pivotal research must focus on comprehending insect physiology to inform biomimetic design and automation strategies. By deepening our understanding of the biological processes at work, we can devise standardized, automated production systems that would enable substantial labor cost reductions over time. Developing these systems requires dedicated research and development to address the complexities of rearing and harvesting insects on a mass scale. Without this targeted investment in innovation, the industry will struggle to progress toward cost-efficient, large-scale operations.

### Inconsistency in quality

The quality of insect products can be affected at three main steps in the production process: the food provided, the time of harvest, and the method of processing larvae into meal. The quality of substrates used for larval cultivation can significantly influence their composition and nutritional value as a feedstuff. This impact manifests both directly, as observed in alterations to larval lipid, protein content and amino acid profiles, and indirectly, through effects on larval growth and maturation rates, including developmental aberrations or delays induced by malnutrition. Likewise, development rate of ectothermic insect larvae is highly dependent on environmental temperature, such that optimal growth rate and harvest time can be assured only in controlled environments. If not adequately provided by the grower, these factors can introduce compositional variability of the larvae that compromises consistency and complicates standardization across different insect farming companies. Implementing strategies that ensure consistent quality of feed stocks (that may need testing and supplementation), and environmental controls, adds a layer of complexity and costs to the production process. These considerations present additional challenges to ensuring outcomes in current larval farming operations and future plans for scale-up. Finally, heat pasteurization of larvae to kill potential pathogens, and/or overheating during drying stages, can reduce the product’s nutritional value. Beyond the proof-of-principle results, comprehensive farm-level aquaculture data collection over time would be important to rigorously assess insect protein-based aquafeed performance and provide feedback to insect producers to further refine their production methods.

### Unknown value of byproducts

While the primary product of insect production is larva meal, other byproducts, such as frass and chitin, also accumulate during the process and are not without value. However, these insect byproducts are novel, and their actual market value is hard to determine in a production context that is yet speculative. A business model that includes secondary revenue streams, in combination with offsetting byproduct disposal costs and downstream environmental impacts, could expand the scope of products leaving insect production facilities. The means of extracting, processing, and transporting these products would need to be cost-effective, arguably justifying the need for further research to help insect producers assess the value of tangential business opportunities.

### Concerns and threat of new problems

Although there are a few studies evaluating the safety of insect meal in terms of contaminants and microbes, there are none on the safety issues surrounding the production process itself. Biosecurity for an operation that is labor intensive can be a critical concern when dealing with decaying waste that might have frequent bacterial and mycotic blooms. Risk of infections, endotoxin/mycotoxin exposure, and allergens that could lead to health problems might need to be addressed. There is already a known concern that the waste substrates attract other species of flies to lay en route to larva cultivation facilities introducing undesirable larvae in the cultivation mix. Such invasive larvae would affect consistency of insect meal quality. Moreover, some of these invasive flies that have a shorter of life cycle emerge prior to harvesting causing potential public health concerns. Addressing these problems at scale without added costs can be a challenge for this industry.

Another core concern is that of any intensive farming operation in that monocultures are susceptible to infections due to high-density populations and confined environments. Factors associated with stress from suboptimal conditions and nutritional issues compounded by reduced genetic diversity might lead to elevated susceptibility and rapid spread of pathogens. Over time, the industry might also see devastating emerging pathogens due to selective pressure and uniform host vulnerability. Therefore, genetics research to maintain populations through stock insect breeding schemes to stably sustain diversity would significantly reduce pathogen risks in the long run.

### Regulatory challenges

The regulation of insect-based ingredients currently varies significantly by region, creating a complex landscape for producers and farmers to navigate. Ensuring compliance with local and international standards is essential for widespread adoption. Production and use of insect meals for animal production is legal in the EU and the United States; however, there remains a lack of certainty regarding quality of substrates/waste streams that can be used for larval cultivation. Moreover, there are other regions outside the EU and the United States that do not seem to have undergone similar regulatory scrutiny. Seafood, however, is a global commodity, with most aquaculture products exported from Asia, often from countries with different agricultural standards than the importing countries. Currently, this does not seem to be an issue but, with traceability and raw materials becoming more of a concern for environmental, labor, and health reasons, it is not out of the question that conflicting food production standards could lead to trade restrictions and significant hurdles for this growing industry.

## Conclusion: The Need for More Research and Caution

In summary, like many forms of agriculture, breeding flies entails maintaining a delicate equilibrium between technological innovation and natural processes through biomimicry. Key to success in this field is the ability to consistently induce mating among captive adults, construct specialized facilities, control operational expenses and fluctuating quality, secure customers open to shifting traditional practices, and invest in technology that enhances productivity and quality. While several companies have entered the scene, the sector still lacks standardized methodologies and best practices, with much of its operations cloaked in secrecy as firms vie for market dominance. Therefore, the need for speed given the broader urge for implementing such a system has not been met with academic engagement and federal support for scientific research on guiding possible improvements to the production process. Moreover, addressing concerns without conflicts of interest need independent agencies and federal incentives that can address and highlight the safety and risks associated with the use of insect-based aquafeed. Nevertheless, the prospect of utilizing insects as a sustainable, high-protein feed for livestock holds great promise, yet overcoming the substantial hurdles that remain is essential for this vision to materialize.

## About the authors

 **Vimal Selvaraj** is a professor of Integrative Physiology in Cornell University’s Department of Animal Science, combines his veterinary background with a deep commitment to enhancing livestock-based food systems sustainably. His work spans fundamental physiological mechanisms and practical farming and management applications, aims to boost farm efficiency. Notably, his research has explored converting dairy waste into housefly larva meal for aquaculture, highlighting the potential of insect-based feeds in promoting One Health. He actively engages in collaborations to refine production methods and investigate the values in insect-based feed. **Corresponding author**: vs88@cornell.edu

 **Eugene Won** is a Sr. research associate in the Animal Science Department at Cornell University, performs aquaculture and fisheries research, teaches courses on fish biology and production, and serves as Academic Coordinator for Shoals Marine Lab. He runs a recirculating aquaculture/aquaponics research lab designed to develop and test novel, sustainable aquafeed systems capable of salvaging nutrients from industrial byproducts (e.g., seafood processing discards and insect larvae) or other liabilities (invasive species). He works across the disciplines to prosper collaborative research relationships with commercial aquaculturists and other seafood producers (fishermen, seafood dealers, and processors), academics and government agencies.
